# Mitochondrial Permeability Transition

**DOI:** 10.3390/cells11233866

**Published:** 2022-11-30

**Authors:** Paolo Bernardi, Evgeny Pavlov

**Affiliations:** 1 Department of Biomedical Sciences, University of Padova, Via Ugo Bassi 58/B, I-35131 Padova, Italy; 2 College of Dentistry, Department of Molecular Pathobiology, New York University, New York, NY 10010, USA

The mitochondrial permeability transition (PT) is a phenomenon that can be broadly defined as an increase in the permeability of the mitochondrial inner membrane. This increase can be caused a variety of physiological as well as pathological stimuli. The PT disrupts the process of mitochondrial oxidative phosphorylation, dramatically affecting mitochondrial function with profound consequences on cellular and organism function. In most cases, the PT is linked to the opening of a large conductance pore: the mitochondrial permeability transition pore (mPTP). This Special Issue, titled “Mitochondrial Permeability Transition”, presents a collection of reviews that discuss the recent progress in investigating the roles played by PT in various pathologies. 

Jia and Du review the experimental evidence on the involvement of the PT in ageing and Alzheimer’s disease [1]. At present, the mechanistic link between the PT and ageing and AD remains elusive. Here, authors’ summarize experimental evidence on the existence of such a link. They argue that mPTP overactivation can be implicated in mitochondrial dysfunction in aging and AD brains. Ten et al. present a comprehensive review on how the activation of the PT contributes to neonatal brain and lung injuries [2]. This review also focuses on the potential mechanistic relevance of the PT in neonatal diseases caused by acute ischemia–reperfusion stress during this early developmental stage. Mironova and Pavlov discuss various aspects of the function and pathophysiological role of a “non-conventional” PT that is caused by the Ca^2+^-induced formation of a lipidic pore by palmitic and stearic acids [3]. They summarize experimental evidence which demonstrates that this lipidic pore might play a protective role against glutamate toxicity. Rottenberg and Hoek discuss how the activation of the mPTP can relate to ageing, disease, and longevity [4]. They suggest that activation of the mPTP is a general phenomenon that can lead to increased autophagy/mitophagy, turning these processes into a destructive event leading to cell aging and death. They conclude that a more comprehensive understanding of the mechanisms underlying mPTP inhibition and activation will lead to the discovery of new drugs that will eventually help to slow aging and degenerative diseases. Finally, Brustovetsky covers the controversial issue of the role played by the adenine nucleotide translocator (ANT) in the formation of the mPTP [5]. He summarizes experimental evidence supporting the idea that the ANT can provide an mPTP channel, and concludes that the mPTP can form from either ANT or ATP synthase depending on specific conditions.

In addition to the review articles, this Special Issue also contains three papers reporting original research relating to the PT. In a mouse model of ALS, Martin et al. report that the therapeutic efficacy of mild whole-body hypothermia is at least partly linked to the inactivation of the mPTP [6]. Readnower et al. used a genetic model of mPTP inhibition—mice lacking the CypD encoding gene *Ppif*—to establish that the mPTP is an important drug target in protection against traumatic brain injury [7]. Finally, Baburina et al. investigated the link between the mPTP and the damage caused by chronic alcohol intoxication [8]. They demonstrate that alcohol exposure leads to activation of the mPTP and that this activation is modulated by PK11195 and PPIX.

We believe that the Special Issue will substantially contribute to the ongoing debate on the nature and pathophysiological functions of the mPTP. We hope that our readership will find it of interest.
